# Fragility Index and Fragility Quotient in Randomized Controlled Trials on Corticosteroids in ARDS Due to COVID-19 and Non-COVID-19 Etiology

**DOI:** 10.3390/jcm10225287

**Published:** 2021-11-14

**Authors:** Maria Vargas, Annachiara Marra, Pasquale Buonanno, Antonio Coviello, Carmine Iacovazzo, Giuseppe Servillo

**Affiliations:** Department of Neurosciences, Reproductive and Odontostomatological Sciences, University of Naples “Federico II”, 80100 Naples, Italy; dottmarraannachiara@gmail.com (A.M.); pasqual3.buonanno@gmail.com (P.B.); antonio_coviello@live.it (A.C.); iacovazzo@tin.it (C.I.); maria.vargas@unina.it (G.S.)

**Keywords:** COVID-19, acute respiratory distress syndrome (ARDS), corticosteroid, pneumonia, fragility index, fragility quotient, randomized controlled trials (RCTs), systematic review, trial sequential analysis (TSA)

## Abstract

Background: The effectiveness of corticosteroids in acute respiratory distress syndrome (ARDS) and COVID-19 still remains uncertain. Since ARDS is due to a hyperinflammatory response to a direct injury, we decided to perform a meta-analysis and an evaluation of robustness of randomised clinical trials (RCTs) investigating the impact of corticosteroids on mortality in ARDS in both COVID-19 and non-COVID-19 patients. We conducted a systematic search of the literature from inception up to 30 October 2020, using the MEDLINE database and the PubMed interface. We evaluated the fragility index (FI) of the included RCTs using a two-by-two contingency table and the *p*-value produced by the Fisher exact test; the fragility quotient (FQ) was calculated by dividing the FI score by the total sample size of the trial. Results: Thirteen RCTs were included in the analysis; five of them were conducted in COVID-19 ARDS, including 7692 patients, while 8 RCTS were performed in non-COVID ARDS with 1091 patients evaluated. Three out of eight RCTs in ARDS had a FI > 0 while 2 RCTs out of five in COVID-19 had FI > 0. The median of FI for ARDS was 0.625 (0.47) while the median of FQ was 0.03 (0.014). The median of FI for COVID-19 was 6 (2) while the median of FQ was 0.059 (0.055). In this systematic review, we found that FI and FQ of RCTs evaluating the use of corticosteroids in ARDS and COVID-19 were low.

## 1. Background

The use of *p* value < 0.05 was introduced as an arbitrary threshold to declare the statistical significance [1]. Finding a *p* value < 0.05 implies that the null hypothesis (i.e., no difference in outcome between groups), should be rejected [2]. In 2016 the American Statistical Association (ASA) encouraged researchers to go beyond the use of *p* value, since a statistical significance did not mean that a scientific finding is true or correct [3]. The use of fragility index (FI) was introduced recently [4]. The FI is calculated by changing the status of patients without an event to an event in the treatment group with the smallest number of events, until the *p* value exceeds 0.05 [4]. Consequently, the FI represents the number of patients responsible for the statistical significance of a trial finding and it is an intuitive measure of the robustness of the RCTs [5]. Studies with a higher FI are considered more robust [4,5] and may assist the clinicians to interpret the results of their findings [6]. Many RCTs in anesthesiology and critical care reported fragile results with inconsistent conclusions [7]. We focused on RCTs regarding two major fields of critical care research such as ARDS and COVID-19 and applied this new statistical methodology to investigate the role of corticosteroids in these two clinical settings. Since ARDS results from a hyperinflammatory response to a direct injury, the host immune response has a key role in the pathophysiology of the disease. Indeed, evidence showed that COVID-19 pneumonia is associated with both hyperinflammation and immunoparalysis [8]. Several therapies aiming to relieve the inflammatory response are being so far evaluated, but strong evidence of benefit is still lacking. Corticosteroids might have beneficial effects in overcoming hyperinflammation in different diseases and could serve as an easily accessible and affordable treatment option. On the other hand, corticosteroids have well-known adverse effects (e.g., delayed viral clearance, opportunistic infections, suppression of the hypothalamic–pituitary–adrenal axis) that may limit their use [9]. Several RCTs showed a beneficial effect of corticosteroids on short-term mortality and a reduction in the need for mechanical ventilation in COVID-19 ARDS but data are too sparse to draw any conclusions [9]. Therefore, we decided to perform a meta-analysis and an evaluation of robustness of RCTs investigating the impact of corticosteroids on mortality in ARDS in both COVID-19 and non-COVID-19 patients.

## 2. Methods

### 2.1. Study Search

We conducted a systematic search of the literature in MEDLINE and PubMed from inception until 30 October 2020 to find RCTs evaluating corticosteroids in COVID-19, since their use became a standard of care in September 2020, while we expanded the search for RCTs using corticosteroids in ARDS until 30 August 2021. We used the mesh terms: (“acute lung injury” OR “acute respiratory distress syndrome” OR “ARDS”) AND (“glucocorticoids” OR “corticosteroid” OR “steroids” OR “methylprednisolone” OR “dexamethasone” OR “hydrocortisone” OR “prednisolone”) (corticosteroids OR dexamethasone OR steroids OR glucocorticoids OR methylprednisolone) AND (COVID OR coronavirus OR SARS-CoV-2). We excluded trials that were not randomized. We applied an English language restriction. We included only published full papers.

### 2.2. Data Extraction and Quality Assessment

Initial selection was performed by screening titles and abstracts by two pairs of independent reviewers (MV and PB, AM and CI). For detailed evaluation, a full-text copy of all possibly relevant studies was obtained. Data from each study were extracted independently by paired reviewers (MV and PB, AM and CI) using a pre-standardized data extraction form. One pair of reviewers (GS and AC) was not informed about authors, journal, institutional affiliation, and date of publication. Data extracted from the publications were checked by another reviewer for accuracy. We used the Cochrane risk of bias tool to assess the quality of study design and the extent of potential bias [5,6] by considering the following domains: random sequence generation, allocation concealment, blinding of participants, personnel and outcomes assessors, incomplete outcome data, selective outcomes reporting, baseline characteristics, and funding resources. Two reviewers (MV and PB) independently used these criteria to assess the quality of trials. We resolved any disagreement by consensus or consulting a third reviewer (AM) if needed.

### 2.3. Qualitative Analysis

Risk of bias was assessed using the Cochrane risk of bias tool for RCTs and the risk of bias instrument for non-randomized studies of interventions (ROBINS-2) [10]. The certainty of evidence was assessed using the GRADE approach [11] taking into consideration, for each outcome, all the factors which might influence certainty. Factors that may reduce the certainty of evidence include risk of bias (study limitations), inconsistency (unexplained heterogeneity across study findings), indirectness (applicability or generalizability to the research question), imprecision (the confidence in the estimate of an effect to support a particular decision) or publication bias (selective publication of studies). The certainty of evidence may be strengthened if the following considerations are present: large or very large magnitude of effect, evidence of a dose-response gradient, or opposing residual confounding. GRADE summary of findings and tables were developed with GRADEpro GDT software (McMaster University, Hamilton, Ontario, USA 2015. Developed by Evidence Prime, Inc. Available at: https://gradepro.org/, accessed on 11 November 2021).

### 2.4. Quantitative Analysis

We evaluated the FI of the RCTs included in this systematic review using a two-by-two contingency table and a *p*-value produced by the Fisher exact test [12]. According to the FI, we defined robust RCTs with FI > 0, and not robust RCTs with FI = 0. The fragility quotient (FQ) is calculated by dividing the FI score by the total sample size of the trial [13]. The FQ provides a method to standardize the fragility to the sample size of a trial. A smaller FQ also indicates a less robust study outcome [13].

The meta-analysis was conducted according to the Preferred Reporting Items for Systematic Reviews and Meta-Analyses (PRISMA) guidelines. Meta-analysis was performed with mixed random effect using DerSimonian and Laird method. Results were graphically represented using a forest plot. The relative risk (RR) and 95% CI for each outcome were separately calculated for each trial pooling data when needed, according to an intention-to-treat principle. The choice to use RRs was dictated by the design of meta-analysis based on RCTs. Tau^2^ defined the between-studies variance. Outcome differences between experimental and control groups were tested using a two-sided Z test and considered statistically significant if *p*-value was less than 0.05. The homogeneity assumption was checked with a Q test with a degree of freedom (df) equal to the number of analyzed studies minus 1. The heterogeneity was measured by the I^2^ metric, which describes the percentage of total variation across studies that is due to heterogeneity rather than by chance. I^2^ was calculated as I^2^ = 100%·(Q − df)/Q, where Q is Cochran’s heterogeneity statistic and df is the degrees of freedom. A value of 0% indicates no observed heterogeneity, and larger values show increasing heterogeneity. Analyses were conducted with OpenMetaAnalyst (version 6) Brown University, Providence RI, USA and SPSS version 20 (IBM, Milan, Italy). To evaluate potential publication bias, a weighted linear regression was used, with the natural log of the RR as dependent variable and the inverse of the total sample size as independent variable. This modification of Macaskill’s test gives more balanced Type I error rates in the tail probability areas in comparison to other publication bias test.

Trial sequential analysis (TSA) depends on the quantification of the required information size (RIS). TSA was undertaken using TSA 0.9 beta software if the number of included trials was more than five. The RIS was estimated using relative risk reduction and heterogeneity adjusted information size for dichotomous outcomes. The result was confirmed as true positive if the cumulative Z-curve surpassed the Lan-DeMets trial sequential monitoring boundary or reached the RIS above the conventional significance level line (Z = 1.96); the result was confirmed as true negative if the cumulative Z-curve reached the futility boundary or reached the RIS below the conventional significance level line (Z = 1.96). TSA adjusted 95% CIs were also presented.

## 3. Results

### 3.1. Study Selection

**Figure 1:**
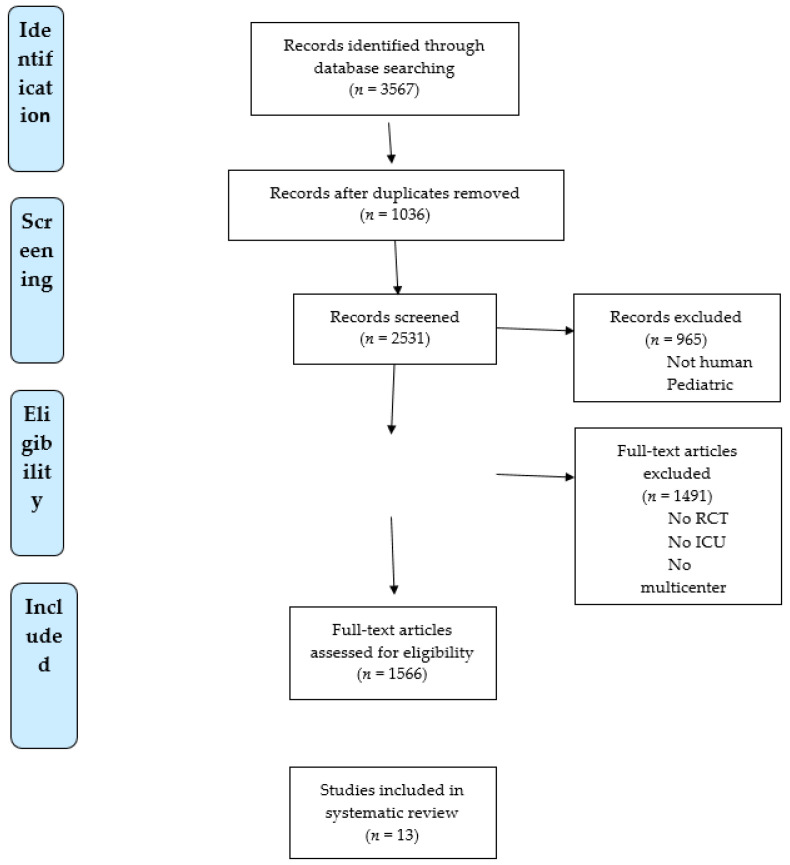
PRISMA flow chart of included studies evaluating the use of corticosteroids in non-COVID-19 and COVID-19 ARDS.

A total of 3567 studies were identified; 1036 of them were duplicates; 1566 full-text articles were assessed for eligibility and 13 RCTs were included in the analysis [14,15,16,17,18,19,20,21,22,23,24,25,26]. Figure 1 shows the flow diagram for included studies. We included the study of Confalonieri et al. on the use of corticosteroids in severe community-acquired pneumonia since the enrolled patients presented a clinical and radiological evidence of pneumonia with bilateral or multi-lobar involvement and a PaO_2_/FiO_2_ ratio less than 250; in particular, the mean PaO_2_/FiO_2_ ratio was 141 in steroid group and 178 in control group. All these characteristics are consistent with those reported by the other studies of our meta-analysis.

**Figure 2:**
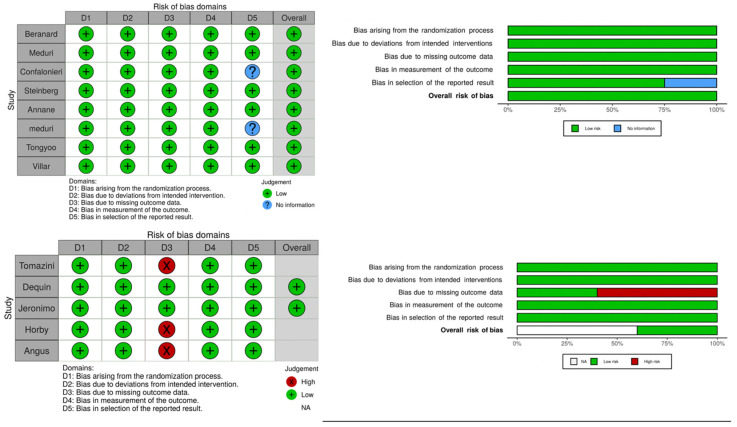
Risk of bias summary. Green represents a low risk of bias, yellow some concerns, and red a high risk of bias.

Corticosteroids in COVID-19 ARDS were evaluated in 5 RCTs including 7692 patients [14,15,16,17,18], while 8 RCTs with 1091 patients evaluated the use of steroids in non-COVID-19 ARDS [19,20,21,22,23,24,25,26]. Low risk of bias was found in all the RCTs investigating the use of corticosteroids in non-COVID-19 ARDS [19,20,21,22,23,24,25,26] while 3 out of 5 RCTs in COVID-19-ARDS had a low risk of bias [15,16,18] (Figure 2). Main characteristics of included studies were reported in Supplementary Materials (Table S1).

### 3.2. Meta-Analysis including All the RCTs

**Figure 3:**
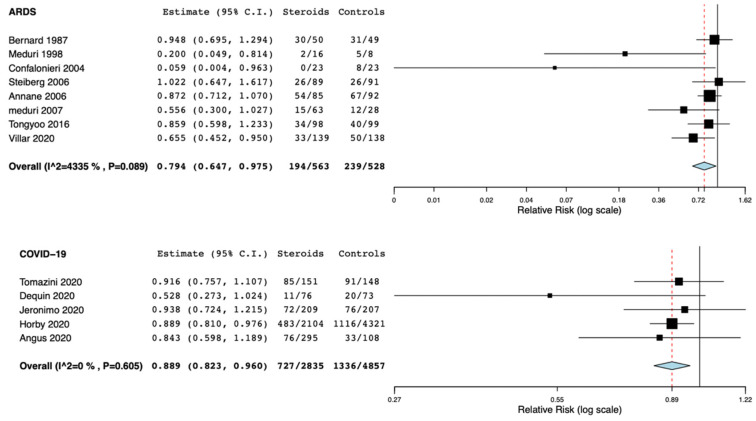
Forest plots for mortality in all included studies evaluating the use of corticosteroids in non-COVID-19 and COVID-19 ARDS. C.I.: confidence interval.

The use of corticosteroids in non-COVID-19 ARDS reduced the risk of hospital mortality (RR: 0.79, 95% CI 0.64–0.97, I^2^ = 43%) (Figure 3). The use of corticosteroids in COVID-19 ARDS reduced the risk of hospital mortality (RR: 0.89, 95% CI 082–0.96, I^2^ = 0%) (Figure 3).

### 3.3. Fragility Index, Fragility Quotient, p-Value

**Figure 4:**
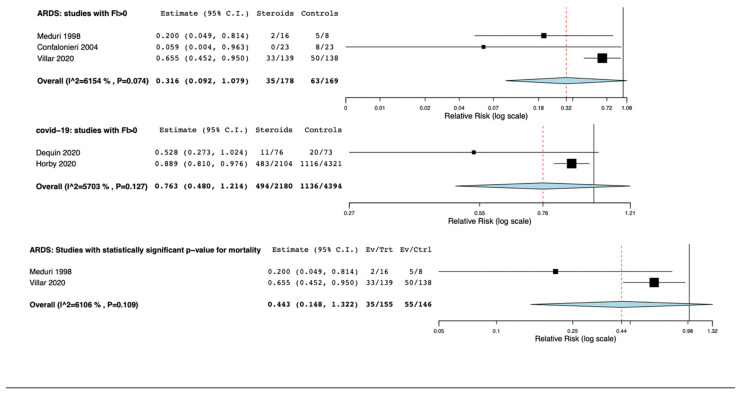
Forest plots for mortality of studies with FI > 0 and statistically significant *p* value evaluating the use of corticosteroids in non-COVID-19 and COVID-19 ARDS. C.I.: confidence interval.

Three out of eight RCTs in non-COVID-19 ARDS had a FI > 0 [19,23,24] while 2 out of 5 RCTs in COVID-19 ARDS had FI>0 [14,16]. The median of FI for non-COVID-19 ARDS was 0.625 (0.47) while the median of FQ was 0.03 (0.014). The median of FI for COVID-19 ARDS was 6 (2) while the median of FQ was 0.059 (0.055). Meta-analyses performed only with the RCTs with FI > 0 showed that the use of corticosteroids did not reduce mortality in both non-COVID-19 and COVID-19 ARDS (RR: 0.31, 95% CI 0.09–1.09, I^2^ = 61%. RR: 0.73, 95% CI 0.48–1.21, I^2^ = 57%, respectively) (Figure 4). Two out of eight RCTs in non-COVID 19 ARDS [19,23] and 1 RCTs out of five in COVID-19 ARDS reported a statistically significant influence of corticosteroids on mortality [14]. In meta-analyses including only RCTs with statistically significant *p*-value for mortality, we found that the use of corticosteroids did not reduce mortality in non-COVID-19 ARDS (RR: 0.44, 95% CI 0.15–1.21, I^2^ = 61%) (Figure 4) while the RR for the one statistically significant RCT in COVID-19 was RR: 0.89 (95% CI 0.81–0.97).

### 3.4. Trial Sequential Analysis including All RCTs

**Figure 5:**
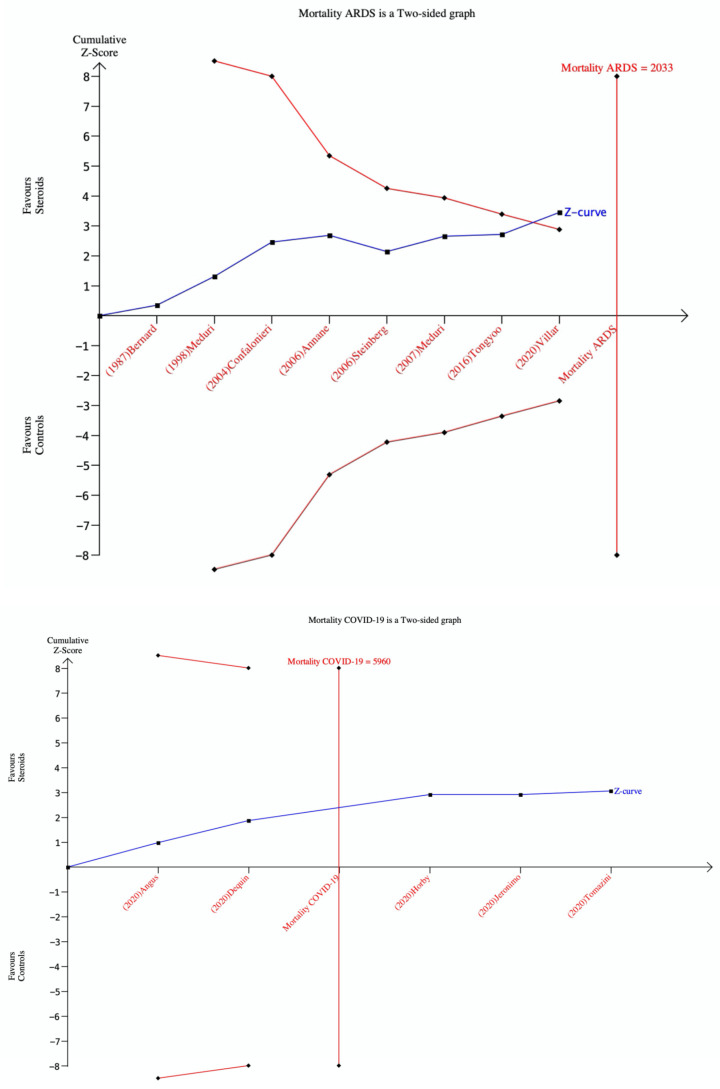
Trial sequential analysis TSA for mortality in all included studies on ARDS (upper box) ND non-COVID-19 ARDS (lower box). TSA was performed with relative risk and random-effects (Der-Simonian and Laird). Zero-event trials are not included.

In the TSA for hospital mortality in non-COVID-19 ARDS, the cumulative Z-curve crossed the Alpha boundary of significance, thus supporting the use of corticosteroids. However, since the cumulative Z-curve failed to cross the TSA boundary and did not reach the RIS of 2033 patients, this result is not conclusive and further studies are needed (Figure 5). In the TSA for hospital mortality in COVID-19 ARDS, the cumulative Z-curve did not cross the alpha boundary of significance, indicating that the results in favor of the use of corticosteroids are not statistically significant. However, since the cumulative Z-curve cross the RIS of 5960 patients, no further studies are needed and the conclusion can be considered definitive (Figure 5).

### 3.5. GRADE Approach

**Figure 6:**
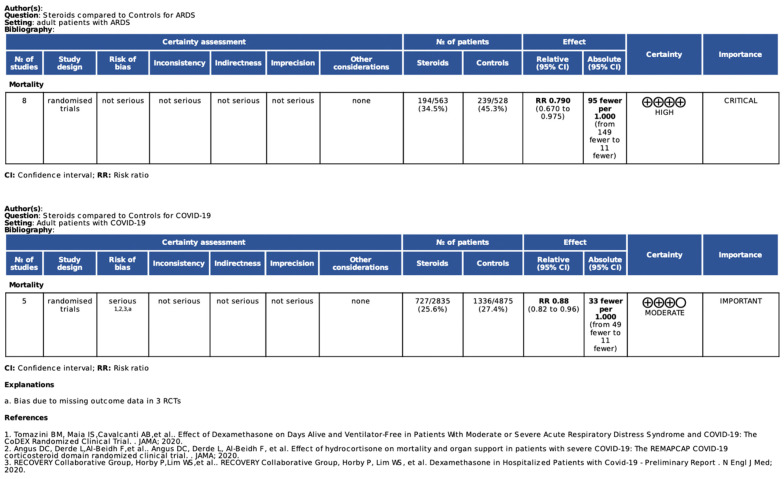
GRADE evidence profile for considered outcome.

Overall evidence was qualified using GRADE for RCTs (Figure 6). High quality of evidence was found for studies investigating the impact of corticosteroids on mortality in non-COVID-19 ARDS patients, while the level of evidence of studies performed in COVID-19 ARDS was downgraded due the high risk of bias mainly caused by missing outcome data.

## 4. Discussion

In this systematic review, including 13 RCTs and 8783 patients evaluating the role of corticosteroids in COVID-19 and non-COVID-19 ARDS, we found that: (1) the median of FI for non-COVID19 ARDS was 0.625 while the median of FQ was 0.059. The median of FI for COVID-19 ARDS was 6 while the median of FQ was 0.022. (2) only three RCTs in non-COVID-19 ARDS and two RCTs in COVID-19 ARDS reached a FI more than zero, (3) only two RCTs in non-COVID-19 ARDS and one RCT in COVID-19 ARDS reached a statistically significant *p*-value for mortality, (4) when performing meta-analysis by including RCTs with a FI > 0 or with a significant *p*-value we found that the use of corticosteroids in both non-COVID-19 and COVID-19 ARDS did not reduce the risk of hospital mortality.

ARDS from all etiology is mostly the result of an innate immune-cell mediated inflammatory response to a direct injury that damages the lung parenchyma [27]. Corticosteroids are powerful anti-inflammatory drugs and may be beneficial in patients with ARDS regardless of etiology [27]. Indeed, a recent meta-analysis indicated that corticosteroids may reduce mortality and duration of mechanical ventilation in all patients with non-COVID-19 and COVID-19 ARDS [27]. According to new evidence, the World Health Organization (WHO) in September 2020, changed its recommendation about the use of corticosteroids supporting the use of systemic corticosteroids for the treatment of patients with severe COVID-19 [28]. This clinical practice guideline was mostly triggered by the results of the RECOVERY trial which included almost the 60% of the population considered in the WHO analysis [14]. The RECOVERY trial among hospitalized patients with COVID-19 showed that use of dexamethasone for up to 10 days resulted in lower 28-day mortality than usual care in patients who were receiving invasive mechanical ventilation at randomization [14,29]. The emergence of those data suggesting corticosteroids improve survival in severe COVID-19, has led to renewed interest in the overall effects of corticosteroids in ARDS; for this reason we performed this study analyzing the RCTs on both COVID-19 and non COVID-19 ARDS.

To our knowledge, this is the first systematic review evaluating the FI and FQ of RCTs to assess the impact of corticosteroids in COVID-19 and non-COVID-19 ARDS. The overall median of FI for non-COVID-19 ARDS studies was low, with 63% of studies with a FI index of 0 while the median of FI for COVID-19 ARDS was 6, with 60% of RCTs with a FI of 0. In the field of critical care RCTs evaluating mortality present a FI of 3 or less in 25% of studies [5]. Furthermore, FI in multicenter RCTs showed a reduced mortality in critically ill patients: eight out of 27 RCTs reporting reduced mortality had a FI equals to 0, 15 RCTs had a FI between 1 and 10, whereas four RCTs had a FI between 11 and 20 [6].

FI is also linked to sample size; it linearly varies with the number of events in the control group while keeping fixed the number of events in the intervention group [30]. This is the reason why FI in studies on COVID-19 ARDS is higher than FI in non COVID-19 ARDS studies, since 6474 patients were e involved in COVID-19 ARDS RCTs with FI > 0 and 347 patients were involved in non-COVID-19 ARDS RCTs with FI > 0.

FI offered a way to test the robustness of statistical significance of RCTs providing an additional perspective to interpret the findings through a frequentist framework [31,32]. The use of the *p*-value approach has been heavily criticized in recent years by the American Statistical Association [3]. Relying on a fixed *p*-value level has been identified as one of the possible causes of the low level of replication rate in current scientific research [31]. Therefore, FI may be a supplementary information along with the *p*-value to provide an intuitive measure of the solidity of RCT the results [31]. In this study, we found that only 2 RCTs in non-COVID19 ARDS and 1 RCT in COVID-19 ARDS had a significant *p*-value for mortality and, interestingly, all of them had a FI more than 0.

In this study, we performed a meta-analysis on mortality by including RCTs with FI > 0. Interestingly, the results of our meta-analysis were not confirmed when only the robust trials were analyzed, probably because the not robust trials drove the results of the overall meta-analysis. Meta-analysis has been extensively used to combine the results of different studies on the same outcome [33] and they are considered one of the most important sources of scientific evidence [34,35]. Since meta-analyses are used to solve uncertainties in research, we strongly suggest performing them by taking into account the FI of each RCT to give additional information about the robustness of the included studies.

Based on FI, we showed that the results of meta-analysis may change by including only robust trials and can be influenced by a small number of events.

This study has several limitations. Although it included RCTs, the number of included studies was small. The included RCTs had different primary end-points, even if all of them reported mortality as outcome. Since the use of corticosteroids in COVID-19 became a standard of care in September 2020 [28] further similar studies were not possible.

## 5. Conclusions

In this systematic review, we found that FI and FQ of RCTs evaluating the use of corticosteroids in COVID-19 and non-COVID-19 ARDS were low, thus indicating that the findings of these studies are not robust. Furthermore, performing meta-analysis by including only RCTs with FI more than zero we found that the use of corticosteroids in both clinical conditions did not reduce mortality.

## Supplementary Materials

The following are available online at https://www.mdpi.com/article/10.3390/jcm10225287/s1, Table S1: main characteristics of included studies. NA: not available, NS: non-significant.

## Abbreviations

FI	Fragility index
RCT	Randomized controlled trial
FQ	Fragility quotient
ARDS	Acute respiratory distress syndrome
TSA	Trial sequential analysis
