# Supplementary material for: Fragility Index and Fragility Quotient in Randomized Controlled Trials on Corticosteroids in ARDS Due to COVID-19 and Non-COVID-19 Etiology
Source: J Clin Med. 2021 Nov 14;10(22):5287. doi: 10.3390/jcm10225287 (PMC8624335; doi:10.3390/jcm10225287)
Supplement: Supplementary file 1 [file jcm-10-05287-s001.zip › jcm-1374386-supplementary.pdf]

Studies in ARDS	N° of patients	Type of corticosteroids	Primary outcome	p-value mortality
Beranard	99	Methylprednisolone		
Meduri	24	Methylprednisolone	Improvement in lung function and mortality.	0.03
Confalonieri	46	Hydrocortisone	Improvement in P/F	0.49
Steinberg	180	Methylprednisolone	Mortality at 60-days	1
Annane	177	Hydrocortisone	28-day survival	NS
meduri	91	Methylprednisolone	1-point reduction in lung injury score (LIS) or successful extubation by day 7.	0.21
Tongyoo	197	Hydrocortisone	28-day all-cause mortality	0.46
Villar	217	Dexamethasone	Ventilator free days at 28-day	0.0047
**Studies in COVID-19**	**N° of patients**	**Type of corticosteroids**	**Main outcome**	**p-value mortality**
Tomazini	299	Dexamethasone	Ventilator free days at 28-day	0.31
Dequin	149	Methylprednisolone	Death at 21 days	0.057
Jeronimo	393	Methylprednisolone	28-day mortality	0.629
Horby	6425	Dexamethasone	28-day mortality	< 0.001
Angus	403	Hydrocortisone	Improvement organ support at 21 days	NA

Table S1: main characteristics of included studies. NA: not available, NS: non significant.
